# An evaluation of the cold chain technology in South-East, Nigeria using Immunogenicity study on the measles vaccines

**DOI:** 10.11604/pamj.supp.2017.27.3.11491

**Published:** 2017-06-23

**Authors:** Angus Nnamdi Oli, Remigius Uchenna Agu, Chibueze Peter Ihekwereme, Charles Okechukwu Esimone

**Affiliations:** 1 Department of Pharmaceutical Microbiology and Biotechnology, Faculty of Pharmaceutical Sciences, Agulu, Nnamdi Azikiwe University, Awka, Anambra State, Nigeria; 2 Biopharmaceutics and Drug Delivery Laboratory, College of Pharmacy, Faculty of Health Professions, Dalhousie University, Halifax, NS, Canada; 3 Department of Pharmacology and Toxicology, Faculty of Pharmaceutical Sciences, Agulu, Nnamdi Azikiwe University, Awka, Anambra State, Nigeria

**Keywords:** Immunogenicity, measles vaccine, childhood immunization, cold-chain technology, South-East Nigeria

## Abstract

**Introduction:**

Vaccines are biological products and their efficacy is affected by storage conditions. They are vital in promoting public health. Failures in immunization programmes often times are blamed on disruption in vaccine cold-chain. This study assessed the immunogenicity/potency of the measles vaccines utilized in childhood immunization in South-East, Nigeria and indirectly assessed the effectiveness of the cold-chain technology in the region.

**Methods:**

This was an experimental study carried out between December 2011 and June 2013. Antibody induction method was used to evaluate the immunogenicity/potency of the measles vaccines sourced from the central cold chain facilities in South-east, Nigeria and indirectly, the effectiveness of the cold chain technology in the zone in maintaining vaccine potency. The neutralizing antibodies in a control group (administered with measles vaccines stored at 37°C for 12 months) and in immunized group were determined after 30 days of immunization using ELISA.

**Results:**

The mean storage temperature of the vaccines at the states vaccines central cold chain facilities was -2.4°C and before storage at study site, it was 5.8°C but at the study site it was -4.54°C. Mean ±Standard Error in the Mean (SEM) IgG titers for the measles vaccines sourced from “Open Market”, Ebonyi, Enugu, Imo, Anambra and Abia States were 0.793±0.051, 1.621±0.015, 1.621±0.015, 1.715±0.081, 1.793±0.051 and 1.683±0.078 respectively while the mean ±Standard Error in the Mean (SEM) IgM titres were 0.857±0.037, 1.400±0.030, 1.391±0.032, 1.339±0.037, 1.405±0.066 and 1.279±0.025 respectively. One way analysis of variance shows that there is statistical difference in the IgG and IgM antibodies titers produced by the control compared to the vaccines (P value < 0.0001). Also, Bartlett's test for equal variances showed that there was statistical difference (P value < 0.0001 for IgG and = 0.036 for IgM). The antibodies elicited by the vaccines from the states were enough to confer protection but the vaccine samples from “Open Market” (control) could not evoke enough antibodies.

**Conclusion:**

The cold-chain technology in the region was judged to be optimal as at the time of vaccine sampling since all the measles vaccines had good immunogenicity profile. However, efforts are still needed to maintain these facilities in good condition in order to ensure effective immunization program in the region.

## Introduction

Vaccines, being a key public health tool in infectious diseases control, require periodic surveillance to affirm efficacy. They are among the first biologics/biopharmaceuticals developed [1]. Childhood vaccines have saved millions of children, with relatively few adverse reactions and will continue to do so if consistent nation-wide immunization programs are carried out [2]. Recently, Nigeria has multiplied efforts to control death due the measles infection [3, 4]. Vaccines, like all thermo-labile biopharmaceuticals, are fragile and require strict temperature-controlled storage conditions for them to be useful in the prevention of target diseases. Keeping thermo-labile biopharmaceuticals properly cooled in extreme climates, during transportation over roads with minimal access, and in locations with poor electricity supply is a major hurdle in developing countries’ health-care system [5, 6]. Necessary tools are needed to protect these vital health products from damage to ensure proper coverage and sustained public health impact. Vaccine cold chain technology employs a cold system that ensures that vaccines stay at the manufacturers’ recommended temperature until administered to a recipient [7]. In most countries, it consists of cold rooms, deep freezers and ice-lined refrigerators, cold boxes, vaccine carriers, ice packs and personnel [8]. It is an indispensable link in any successful immunization programme and the lifeline of all thermo-labile health products. It is essential for safe transportation of these products from the manufacturer through to the place of storage and finally to the place of immunization.

Only two reports so far in the country [5, 9] exist on the assessment of cold chain system. The first was in South-west and the second was in South-east. The instruments used in both studies were site inspection, interviews and questionnaires, while the study sites were the immunization centers/facilities. The studies found some lapses in the cold chain management, with emphasis on vaccines without Vaccine Valve Monitor label, in the immunization facilities/centers visited. The studies also emphasized regular training of the staff in the cold chain facilities visited. It is not yet known the effectiveness of the central vaccines cold chain facilities (CVCF) in keeping the vaccines in proper conditions. The CVCF feed the immunization centers, store large volumes of vaccines and for a longer period than the centers. Also, some immunization centers collect from the central cold chain facilities vaccines that will be just enough for each immunization outreach because of inadequate cold chain facility in the centers. A recent study revealed that ’’weak measles case-based surveillance in some areas, lack of awareness about the disease among parents, vaccine stock-out and lack of adequate cold chain equipment to preserve the vaccine in remote hard-to-reach areas’’ had all contributed to measles outbreaks in some part of Nigeria [10].

The present study aims at using animal model to assess the potency/immunogenicity of measles vaccines sourced from the central cold chain facilities in each of the five states in South-East, Nigeria. The outcome of the study may provide insight on how effective the vaccine cold-chain system is within the region. If the measles vaccines sourced from there could evoke sufficient antibodies to confer protection, then the cold chain system may be assumed to be effective.

## Methods

### The study area

The South-East geopolitical zone of Nigeria has a population of over sixteen million [11] and is comprised of 5 states (Abia, Anambra, Ebonyi, Enugu and Imo). The zone hosts nine tertiary health institutions, several General hospitals and over 400 Primary Health Centers (PHC). Each of these facilities is involved in childhood immunization. A central vaccine cold chain facility is located in the capital city of each state and services the immunization centers in the state. Each of the central vaccine cold chain facility is equipped with varied cold chain technologies (ice-lined refrigerators, deep freezers, Vaccine Valve monitors, regular and uninterrupted source of electricity, etc.). Immunization centres, on receiving vaccines from central vaccine cold chain facility, may store the vaccine briefly before or during vaccine use.

### Collection and storage of the measles vaccines

Samples (2 vials from each State) were collected from CVCF and ’’Open Market’’ at different periods from December 2011 – June 2013 and transported in specially insulated carriers (containing ice packs) designed to optimally maintain temperatures. The months of vaccines collection were guided by vaccine availability and convenience. After receiving of sample from CVCF, they were deposited within 4h at the immunization facility of Nnamdi Azikiwe University Teaching Hospital, Nnewi of Anambra State. Sample vaccines were stored at -4.54 ± 0.36°C in the facility.

### The Animal model used

Sixty albino mice (18-30 g) housed under standard conditions (temperature 26 ± 2°C; relative humidity 45 ± 2%) in the animal house of the Faculty of Pharmaceutical Sciences, Nnamdi Azikiwe University, Agulu were used for the experiments. They had unrestricted access to standard pellet diet and water. The experiments were conducted in the Pharmacology and Toxicology Laboratory of the Faculty of Pharmaceutical Sciences, Nnamdi Azikiwe University Agulu and in the Chemical Pathology laboratory of Nnamdi Azikiwe University Teaching Hospital, Nnewi. The Ethical approval for the study protocol was granted by the Ethics Committee of Nnamdi Azikiwe University Teaching Hospital, Nnewi.

### Selection of the immunization dose

The method suggested by Arciniega & Dominguez-Castillo [12] was used with some modifications. In brief, volumes (0.1, 0.2, 0.4, 0.8 and 1.0 ml) of measles vaccines were injected into mice (n=2)by intra-peritoneal route. The animals were bled 28 days post-immunization and antibody titers estimated using ELISA kit (RD-Ratio Diagnostics Frankfurt, Germany). From the result, 0.05 ml was administered to each mouse to elicit immune responses. This dose fell within the linear portion of the dose-response curve (slope > 0) and maximizes detection of differences in antigen quantity.

### Antibody induction, bleeding of the animals and serum extraction

All experiments involving the samples were conducted within 28 days of vaccine arrival. Vaccines sourced from ‘’Open-market’’ and previously stored at 37°C for 12 months were used as negative control while the pre-packaged positive controls which is a component of the ELISA kit (by RD-Ratio Diagnostics Frankfurt, Germany) was used as positive control. Six groups (n = 10) of Albino mice were immunized with 0.05 ml of the test vaccines by intra-peritoneal injection. Each central vaccine cold chain samples and the “Open-market” samples was tested on a group. The animals were allowed free access to water and food throughout the study period and their cages cleaned daily. Routine daily checks were done on the animals to observe for signs of abnormalities. On the 30th day post-immunization, the animals were bled using heparinized capillary tube inserted just below the eyeball. Their bloods were collected in sterile eppendoff tubes, allowed to clot and then centrifuged at 2,683.2 g for 10 minutes. The sera were carefully pipetted out, transferred into another sterile eppendoff tubes and preserved by freezing at -20°C until ready to use.

### Quantitation of the neutralizing antibody

#### Assay procedure

All reagents and samples of the ELISA kits used for IgG and IgM quantitation were brought to room temperature before use. The preparation of reagents and assay protocol was as directed by the manufacturer of the ELISA kit. In brief, before use, each serum sample was first diluted by adding 10µl to 1ml of sample diluent. Then 100µl of each diluted serum sample and ready to use controls were pipetted into the appropriate wells of the micro-well plate coated with purified and inactivated Measles antigen, leaving the first well for the blank. The plate was incubated for 45 minutes at 37°C. The wells were aspirated and washed 4 times for 30 seconds with 300µl/well washing solution using automatic microplate washer (Stat Fax – 2600, model #: H009775). After blotting and drying by inverting the plates on absorbent material, 100 µl of Enzyme-labelled second antibody conjugate (composed of anti-mouse antibodies labelled with horseradish peroxidase (Sigma-Aldrich, Germany)) were added into the wells and incubated for another 45 minutes at 37°C. The wells were re-aspirated, re-washed 4 times, re-blotted and re-dried followed by the addition of 100 µl Tetramethylbenzidine (TMB) Chromogenic solutions. After incubation for 15 minutes at room temperature, 100 µl of stopping solution was added to each well and the absorbance read at 450nm using Stat Fax - 2100 microplate reader (manufacturer – Awareness Technology, USA). The results are recorded as a ratio of the absorbance of the serum samples and that of the cut-off (the reagent control standard). Report is shown as mean ± standard error in the mean. All standards and serum samples were tested in duplicate.

### Data analysis

GraphPad Prism version 5.00 for Windows, GraphPad Software, Inc. San Diego California USA, [13] was used for data analysis and graphical presentations of results. One-Way Analysis of Variance (ANOVA) was used to check for mean difference across the different treatment groups (‘’open market’’, Ebonyi, Enugu, Imo, Anambra and Abia States), Bartlett's test for equal variances was used to check for difference in variance across the different treatment groups while Dunnett’s Tests of Multiple Comparison was used to check for mean difference between the vaccines sourced from ‘’open market’’ and the test samples. All P values reported are for a two-tailed test. The significance level was chosen at α = 0.05. All data were presented as Mean±SEM.

**Ethical approval and compliance with ethical standards:** the work described in this article was approved by the Ethics Committee of Nnamdi Azikiwe University Teaching Hospital, Nnewi (Approval #: NAUTH/CS/66/Vol.4/220). All animal experiment was conducted in compliance with National Institutes of Health (NIH) guidelines for care and use of laboratory animals. **Funding:** African Doctoral Dissertation Research Fellowship award offered by African Population and Health Research Center (APHRC) in partnership with the International Development Research Centre (IDRC): Grant #: ADDRF Award 2012-2014 ADF 020 and The Canadian Commonwealth Scholarship Program (CCSP) administered by the Canadian Bureau for International Education (CBIE) with funding from the Government of Canada’s Department of Foreign Affairs and International Trade (DFAIT) funded the research. The States’ ministries of Health donated the vaccines.

## Results

Table 1 shows the temperatures of the vaccines at central vaccine cold chain facilities, after transport, and at study storage site. The Table shows that the longer the transit time, the warmer the samples became. The recommended storage temperatures (≤ 0^⁰^C) were maintained at the central vaccine cold chain facilities and at the storage site but not during transportation. However, it was observed that the vaccine vials monitors were still in the Stage 1 condition just before storage. The vaccines sourced from the open market had elevated temperatures far beyond the recommended temperature.

**Table 1:** Temperature conditions of the vaccines

Vaccines Sampling Sites	Measles Vaccines		
	Temperature (⁰C) at collection	Temperature (⁰C) before storage	Temperature (⁰C) at storage
Enugu State	-2	5	-4.5
Ebonyi State	-2	7	-4.5
Imo State	-3	6	-5.0
Anambra State	-3	4	-4.7
Abia State	-2	7	-4.0
Mean ± SD	-2.4±0.55	5.80±1.30	-4.54±0.36
Control (“Open Market’’)	20	8	-4.0

Table 2 shows the lot numbers and validity periods of the sample vaccines used for the study. The vaccines were within their shelf-lives (validity periods) when the study was carried out. The inner squares of the vial monitors of all the vaccine samples were lighter than their outer circles. However, the vaccine vialmonitors of the samples from the Cold-chain Stores in the States were in the Stage 1 condition while the one from the open market was in the Stage 2 condition. Table 3 shows the antibody titers for protective immunity, doubtful Protection and no protection (Too low or No Antibody) as interpreted by the ELISA kit manufacturer. Ratios greater than 1.1 is read as the presence of protective antibody (high antibody titres) while ratios less than 0.9 signify that there is too low protective antibody or none at all. Doubtful protection is recorded at ratios 0.09 - 1.00 and mean that test is inconclusive and as such, should be repeated. A repeat result is judged as no protection.

**Table 2:** The measles vaccines studied

Vaccine	Lot #	Labelled expiration date/Shelf life	Source	Status of the VVM at point of collection	Status of the VVM at immunization/use
Measles	2502512	April 2014	Ebonyi and Enugu States	Inner square lighter than outer circle (Stage 1)	Inner square lighter than outer circle (Stage 1)
	004N2102	Nov 2014	Imo, Anambra, Abia States	Inner square lighter than outer circle (Stage 1)	Inner square lighter than outer circle (Stage 1)
	2502512	April 2014	Control (“Open Market’’)	Inner square lighter than outer circle (Stage 2)	Inner square darker than outer circle (Stage 2)

**Table 3:** ELISA Kits’ manufacturer’s interpretative values

Antibody Values	Status
> 1.1	Protective Antibody
– 0.9 < Value < 1.1	Doubtful Protection
Values < 0.9	No Protection (Too low or No Antibody)

**Figure 1:**
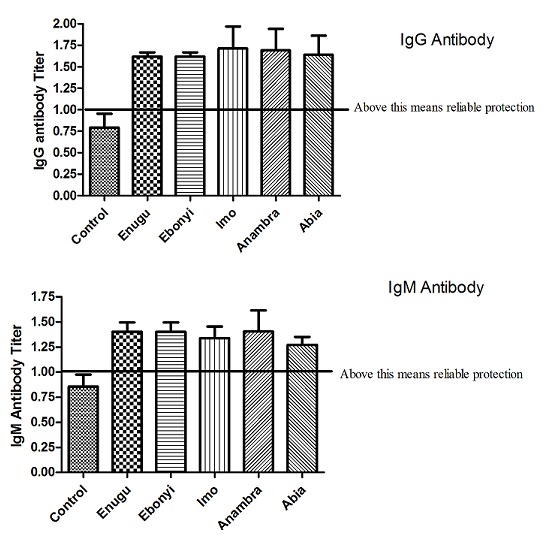
Measles vaccine antibodies titres in the immunized mice

Figure 1 is a graphic presentation of the IgG and IgM antibodies produced by the test vaccines and the negative control. There were significant differences in the IgG antibody titres (P value < 0.0001) as shown by One-way analysis of variance and Bartlett's test for equal variances. Dunnett's Multiple Comparison Test for the IgG antibody titers showed that there were significant differences between the control and the test vaccine samples with 95% confidence interval of differences of -1.042 to -0.6119 (Enugu), -1.042 to -0.6119 (Ebonyi), -1.137 to -0.7061 (Imo), -1.115 to -0.6840 (Anambra) and -1.060 to -0.6297 (Abia). The differences in the mean IgG antibody titres of the control and the individual vaccines from the states were -0.8272 (Enugu), -0.8272 (Ebonyi), -0.9213 (Imo), -0.8993 (Anambra) and -0.8449 (Abia).

The results of inferential statistics of the IgM antibody titers were similar to the IgG titer except that the p values differ slightly. P values were < 0.001 for One-way ANOVA and = 0.0386 for Bartlett's test for equal variances. Dunnett's Multiple Comparison Test for the IgM antibody titers also showed significant differences between the control vaccines and sample vaccines with a 95% confidence interval of differences of -0.6892 to -0.3970 (Enugu), -0.6892 to -0.3970 (Ebonyi), -0.6274 to -0.3352 (Imo), -0.6938 to -0.4015 (Anambra) and -0.5613 to -0.2691 (Abia). The differences in the mean IgM antibody titres of the control and the individual vaccines from the states were -0.5431 (Enugu), -0.5431 (Ebonyi), -0.4813 (Imo), -0.5476 (Anambra) and -0.4152 (Abia).

## Discussion

Huge sums of money are invested in the procurement of vaccines and cold-chain facilities and their maintenance. The goal is the administration of potent vaccines that will evoke the desired immunopharmacological responses that will protect recipients from target diseases. To this end, a good and effective maintenance of cold chain will significantly maintain vaccine potency, safety and immunogenicity profile throughout its shelf-life.

Transport situation would affect temperature of the samples due to a number of reasons. These may include the vaccine carriers, environmental temperature, and the economic situation in the country. Vaccine carriers used do not have a cooling system but depends on ice-packs studded within the carrier to slow down the rate of loss of heat. The poor economic situation would not allow the low-income earners who are predominantly involved in this job to enjoy the “luxury” of a decent public transport. Besides most of the vehicles in use do not have a functional air-conditioning system. A temperate environmental temperature would not allow temperature increases. Similar results were found in a rural district in India [14].Close monitoring of temperatures is necessary to maintain vaccines at recommended freezing temperature range [15]. Analyses of the antibody titres using inferential statistics show that the levels of protection or amount of antibodies induced by the vaccines differ a lot from those produced by the negative control. The results show that all the test vaccines from central cold chain facilities produced enough antibodies to confer protection. This suggests that the cold-chain technology in the vaccine central cold chain facilities in the states are effective and may explain why the zone is relatively free from measles infection except some few reported cases [16]. They attributed the cases to declined vaccine coverage in the affected state and not to cold chain issues. It is established that effective cold chain maintenance and consistently sustained 100 % immunization coverage can actually eradicate infectious diseases like measles [6, 16]. Measurement of measles neutralizing antibodies that confers protection post-vaccination is currently the surrogate marker of measles vaccine efficacy [17, 18]. Through micro-neutralization test, it had been shown that the correlate of protection induced by vaccination using measles vaccine is not necessarily the same correlate that operates to prevent infection [19]. The study [19] also showed that antibody titers >200 mIU/mL after vaccination are protective against infection, but titers between 120 and 200 mIU/mL protect against clinical signs of the disease but not against infection. Also, titers < 120mIU/mL do not produce immunity at all. A more recent work revealed that “the development and magnitude of the adaptive immune response following measles immunization is directly dependent on the number of plasmacytoid dendritic cells of the innate immune response“ [20]. In 2-dose measles-mumps-rubella (MMR) vaccine coverage, a third dose of MMR vaccine had been demonstrated to be safe and important in fighting and preventing measles outbreak [21]. A study in Abia state [22] showed the need to use measles-mumps-rubella (MMR) vaccine in place of measles vaccines alone during childhood immunization. Safe and effective vaccination programme is, therefore, a function of many factors especially effective cold chain maintenance [8, 23].

## Conclusion

The measles vaccines from the central vaccine cold chain facilities were judged to be within the expected potency and immunogenicity margin. This indicates a functional and effective cold-chain system at the central vaccine cold chain facilities in South-East Nigeria at the time of vaccine sampling. However, efforts are still needed to maintain these facilities in good condition in order to ensure effective immunization program in the region.

### What is known about this topic

- Vaccines efficacy is affected by storage conditions;
- Vaccines are vital in promoting public health;
- Disruption in vaccine cold-chain promotes immunization failures and vaccine wastage.

### What this study adds

- We evaluated the immunogenicity of the measles vaccines obtained from central cold-chain facilities in South-eastern Nigeria using animal model;
- We demonstrated that the vaccines were potent and by implication, the central cold chain technology in South-East, Nigeria was perfect at the time of vaccines sampling;
- We demonstrated that good cold chain technology can maintain vaccine’s immunogenicity profile up to the shelf-life.
- We demonstrated that continuous monitoring of the efficiency of the cold-chain technology and vaccine potency testing will bring success in childhood immunization program in the country.

## Competing interests

The authors declare no competing interest.
